# Fairness: from the guts to the brain – a critical examination by Atlantic fellows of the Global Brain Health Institute

**DOI:** 10.3389/fpsyg.2023.1241125

**Published:** 2023-10-19

**Authors:** Thiago Junqueira Avelino-Silva, Natalia Trujillo, Chinedu Udeh-Momoh

**Affiliations:** ^1^Atlantic Fellowship in Equity in Brain Health, Global Brain Health Institute, University of California, San Francisco, San Francisco, CA, United States; ^2^Laboratorio de Investigacao Medica em Envelhecimento LIM66, Servico de Geriatria, Hospital das Clinicas HCFMUSP, Faculdade de Medicina, Universidade de São Paulo, São Paulo, Brazil; ^3^Mental Health Research Group, National School of Public Health, University of Antioquia-UDEA, Medellin, Colombia; ^4^Stempel College of Public Health and Social Work, Florida International University, Miami, FL, United States; ^5^Ageing Epidemiology Research Unit, School of Public Health, Imperial College London, London, United Kingdom; ^6^Centre for Healthy Brain Aging, Brain and Mind Institute, Aga Khan University, Nairobi, Kenya; ^7^Division of Clinical Geriatrics, Karolinska Institute, Stockholm, Sweden; ^8^Wake Forest University School of Medicine, Winston-Salem, NC, United States

**Keywords:** fairness, equity, social justice, brain health, neurobiology

## Abstract

In January 2023, the Global Brain Health Institute (GBHI) at UCSF hosted an online salon to discuss the relationship between fairness and brain health equity. We aimed to address two primary questions: first, how is fairness perceived by the public, and how does it manifest in societal constructs like equity and justice? Second, what are the neurobiological foundations of fairness, and how do they impact brain health? Drawing from interdisciplinary fields such as philosophy, psychology, and neuroscience, the salon served as a platform for participants to share diverse perspectives on fairness. Fairness is a multifaceted concept encompassing equity, justice, empathy, opportunity, non-discrimination, and the Golden Rule, but by delving into its evolutionary origins, we can verify its deep-rooted presence in both human and animal behaviors. Real-world experiments, such as Frans de Waal’s capuchin monkey study, have proven enlightening, elucidating many mechanisms that have shaped our neurobiological responses to fairness. Contemporary cognitive neuroscience research further emphasizes the role of neuroanatomical areas and neurotransmitters in encoding fairness-related processes. We also discussed the critical interconnection between fairness and healthcare equity, particularly its implications for brain health. These values are instrumental in promoting social justice and improving health outcomes. In our polarized social landscape, there are rising concerns about a potential decrease in fairness and prosocial behaviors due to isolated social bubbles. We stress the urgency for interventions that enhance perspective-taking, reasoning, and empathy. Overall, fairness is vital to fostering an equitable society and its subsequent influence on brain health outcomes.

## Introduction

Fairness plays a vital role in shaping human experiences across various aspects of life (Scarpa et al., 2021). Recognizing its importance, the Global Brain Health Institute (GBHI) has identified fairness as one of its core values, emphasizing its significance in creating a just and equitable society. In this context, fairness is defined as the just and unbiased treatment of all individuals, regardless of their background, guided by principles of justice and equality. Our activities were framed within the theoretical understanding of social justice theories, which emphasize the role of fairness in equitable resource distribution and social inclusion.

In January 2023, three Atlantic Fellows from the GBHI at the University of California San Francisco (UCSF) organized an online salon titled “Fairness: from the guts to the brain,” which aimed to explore and critically assess the value of fairness and its relation to brain health and equity. An online salon refers to a virtual gathering of individuals who engage in intellectual discussions and exchange ideas on a specific topic. This format enables participants from various locations to come together, fostering a diverse and inclusive environment for deep and meaningful conversations.

In the online salon, participants critically examined the concept of fairness, discussing its primal component and exploring the instinctive, visceral reactions to unfairness. However, the salon also emphasized that promoting fairness requires more than just gut feelings. Immediate reactions can lead to biased or unfair actions, necessitating active reflection on fairness as a concept and intentional efforts to cultivate it as a value. Throughout the salon, the value of fairness was analyzed in depth, considering its manifestation within various cultural and community contexts and delving into the science behind it. By engaging in this critical examination, participants gained a deeper understanding of the complexities surrounding fairness and its role in fostering a more just and equitable society.

In the following sections, we first investigate personal views on fairness. Then, we examine its evolutionary roots in humans and animals. We move on to its neurobiological basis, guided by cognitive neuroscience research. We also discuss its vital role in healthcare equity and brain health, incorporating diverse cultural insights. Finally, we consider the future of fairness in a polarized world.

### Exploring the multifaceted concept of fairness: insights from public opinion

Fairness is a concept that pervades various aspects of human life and is deeply ingrained in our moral, social, and political values (Schroeder et al., 2019). To understand what people think about fairness, we conducted a two-step investigation, collecting word cloud responses during our online session and taking to the streets of San Francisco to gather first-hand opinions.

**Figure 1:**
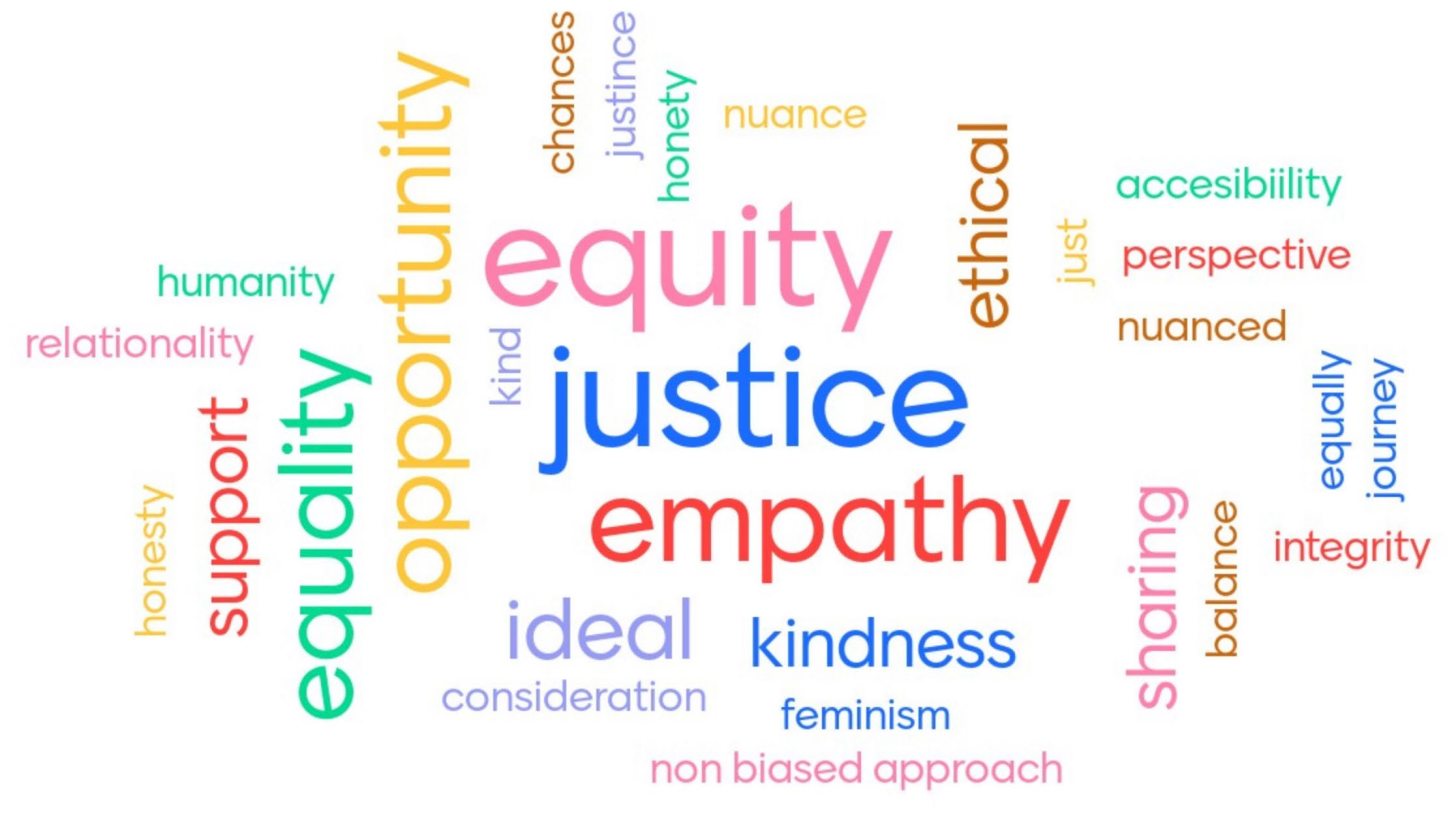
Word Cloud on the salon participants’ responses to “What words come to your mind when thinking on the concept of Fairness?”. The size of each word in the cloud represents its frequency as a response. Larger words were mentioned more often in the public opinion survey (created using mentimeter.com).

To gain an initial grasp of people’s thoughts on fairness, we asked our 40 salon participants (Atlantic Fellows for Equity in Brain Health, GNBHI, and UCSF professionals) to provide one or a few words that they associate with the concept. The resulting word cloud revealed four prominent terms: equity, justice, empathy, and opportunity (Figure 1). These findings suggest that fairness, in the public’s perception, revolves around the ideas of equal treatment, the application of just principles, the ability to understand and share others’ feelings, and the provision of chances for individuals to succeed.

To further explore public opinion on fairness, we also approached eight anonymous individuals of different ages and backgrounds in the streets of San Francisco to capture more nuanced and diverse perspectives. The responses were varied but revealed some recurring themes:

1. Fairness as non-discrimination: Participants emphasized the importance of not judging people based on race, gender, religion, or other traits but instead on their character.
2. Fairness as enabling opportunity: Respondents noted that fairness meant giving everyone opportunities and helping people explore those opportunities, including having a political voice and access to education, jobs, and housing.
3. Fairness as emotional warmth: Some interviewees highlighted the emotional aspect of fairness, associating it with feelings of warmth and kindness.
4. Fairness as social justice: Many responses pointed to broader social issues, such as the Black Lives Matter movement, indicating that fairness is closely connected to the pursuit of social justice.
5. Fairness as the Golden Rule: A particularly poignant testimony from a formerly homeless individual emphasized the importance of treating others as one would like to be treated (i.e., the Golden Rule, an ethical principle found in many cultures and religions), encapsulating the essence of fairness.

It is important to note that the activities described were not designed as formal research with systematic methods and analysis plans. Rather, they were illustrative activities aimed at enriching an educational discussion on fairness. As our interviews were conducted in San Francisco, United States, one must consider that perceptions of fairness may vary based on demographic factors such as cultural background, age, and socioeconomic status. Our observations, therefore, may not be universally applicable and could differ in other settings. Properly designed research studies could explore how these demographic variables influence public perceptions of fairness. Even so, our exploration has suggested a multifaceted concept encompassing equity, justice, empathy, opportunity, non-discrimination, and the Golden Rule. By understanding these diverse dimensions of fairness, we can better engage in meaningful conversations and strive to create a more just and equitable society.

### The evolutionary origins of fairness

Building upon this multi-dimensional understanding of fairness, as reflected in public opinion, we delve deeper into the evolutionary origins of this essential concept. The perception of fairness is not limited to humans but is also observable in certain animal species, particularly those that engage in collective hunting. Once the target is achieved, rewards, typically in the form of food, should be distributed proportionally to the effort each individual has contributed. This equitable share is vital for replenishing energy and maintaining group harmony. Failure to distribute rewards fairly may lead to aggressive behavior or the death of individuals who have exerted significant effort. Moreover, unfair distribution of rewards may lead to the exclusion of individuals from the group (Brosnan and De Waal, 2014).

In the human context, the development of language has further refined our ability to make fairness-related decisions over the long term (Brosnan and De Waal, 2014; McAuliffe et al., 2017). Language has empowered societies to achieve more complex goals and has allowed the integration of community values, such as charity, into the decision-making process. While further research is needed to fully comprehend the impact of language on fairness, charitable actions are not only morally commendable but can also carry economic incentives, such as tax benefits. These economic factors can further influence decision-making, thereby emphasizing the societal importance of fairness.

Incorporating abstract thinking into decision-making processes has been pivotal in understanding fairness as a form of secondary compensation (Brosnan and De Waal, 2014; McAuliffe et al., 2017). Imagine a local community organizer who advocates for the creation of a community garden in a low-income neighborhood. The immediate costs and labor involved might seem like a burden to some community members. However, employing abstract thinking allows the group to consider secondary compensations: the garden could serve as a source of fresh produce, a learning environment for children, and a communal space that fosters neighborhood cohesion. Over time, these benefits could even contribute to lower crime rates and improved mental health among residents. In this example, abstract thinking enables individuals to weigh the immediate costs against a broader range of long-term benefits, thereby promoting fairness in the form of secondary compensation. Abstract thinking enables the evaluation of even more intricate cost–benefit scenarios, taking into account local, regional, or even global objectives such as those outlined in the sustainable development goals agenda.

### The neurobiology of fairness

#### Exploring parallels between human and animal reactions to inequity

The neurobiology of fairness has become a subject of interest among researchers, as understanding the biological basis of our reactions to unfairness may provide valuable insights into human behavior and social structures. With that in mind, we conducted an experiment with our salon participants to explore their reactions to unfair situations. We then drew parallels between these human reactions and those observed in animals, specifically by examining Frans de Waal’s famous study involving capuchin monkeys receiving unequal pay (Brosnan and de Waal, 2003).

In our social experiment, salon participants were electronically grouped with two non-player characters and assigned unique avatars (Supplementary Appendix A). The objective was to reach the end of a designated path, with the first two players securing job opportunities while the third remained unemployed. Participants answered questions related to fairness at each step, which allowed them to move forward. The game was designed to simulate real-world unfairness, incorporating challenges such as unequal starting positions, limited access to quality food and transportation, and appearance-based discrimination. All real-life participants were covertly set up to experience the series of unfair disadvantages, although they were unaware of this manipulation. For example, they started the game four positions behind due to ‘bonus’ points arbitrarily given to non-player characters. Additionally, they lost a turn because of abdominal pain caused by eating at a low-quality restaurant and not having medical care, thereby spotlighting the issue of healthcare inequities. These intentionally engineered setbacks led participants to report feelings of frustration and irritation, allowing them to empathize with individuals who face similar systemic barriers in real life.

To draw parallels between human and animal reactions to unfairness, we showed an excerpt from Frans de Waal’s TED Talk (De Waal, 2011). The talk featured a capuchin monkey experiment where unequal rewards led to visible agitation. In the experiment, the monkeys were placed in separate but adjoining cages, visible to each other, and trained to exchange a small stone for a cucumber slice as a reward. Both monkeys willingly performed the task when they were rewarded equally. However, when one monkey was given a more desirable grape as a reward instead of a cucumber slice, the other monkey, who continued to receive a cucumber, quickly became agitated. This monkey refused to accept the cucumber and sometimes even threw it back at the experimenter. The demonstration of agitation and refusal to accept the unequal reward showcased the innate sense of fairness in these primates, highlighting a deep-rooted biological reaction to unfairness.

The social experiment conducted with our salon participants and the comparison to Frans de Waal’s capuchin monkey experiment highlight the similarities between human and animal reactions to unfairness. Like the monkeys, our participants displayed emotional responses when faced with unfair situations, highlighting the deeply ingrained neurobiological basis for fairness that transcends species boundaries.

Other studies have shown that humans are not the only species to display complex prosocial behaviors like helping and sharing. Empathy and satisfaction often motivate such actions in humans, and previous research has shown similar behavior in chimpanzees. In a study conducted by Horner et al. (2011), researchers investigated the prosocial choices of chimpanzees using a token-based experimental setup. The chimpanzees were tasked with selecting tokens, with each color representing a different outcome. When the chimpanzees chose one color, they alone received a reward, while selecting the other color resulted in both themselves and their partner receiving a reward. The results demonstrated that when a partner was present, the chimpanzees were more inclined to choose the token that benefited both parties, showcasing their prosocial behavior. This study highlights the connection between negative responses to inequity and cooperation levels across various species. The ability to detect and react to unfairness, or inequity aversion, may have provided an evolutionary advantage by enabling individuals to assess the value of their cooperative partners more accurately.

In summary, both humans and chimpanzees have been observed to respond negatively when they receive more than their partners, suggesting that they are capable of prioritizing long-term cooperative relationships over immediate gains. By adopting a comparative approach, researchers have gained valuable insights into the origins of inequity responses, which in turn deepens our understanding of the human perception of fairness. However, it is worth noting that these observations are generally made within small, cohesive groups. In larger societal contexts where different groups compete for limited resources, survival instincts and competition may take precedence (Lee et al., 2018). While there is limited research on this specific aspect, it raises important questions about the scalability of these prosocial behaviors in more complex social structures.

#### The neural basis of fairness

Cognitive neuroscience research has begun to explore how the human brain encodes fairness-related processes. Most research in this area falls under the umbrella of Decision Neuroscience, an interdisciplinary field that aims to understand the fundamentals of human decision-making (Li and Tracer, 2017). Within this approach, tasks are often designed to prompt participants to decide about monetary divisions in interactive settings, balancing rewards and cooperation with partners. Combined with brain imaging methods, these tasks lead to numerous findings on the neural underpinnings of fairness behavior.

There are several neural mechanisms involved in these complex abstract analyses. For instance, the anterior insula plays a significant role in monitoring fairness or unfairness, becoming more active when someone is faced with an unfair situation (Li and Tracer, 2017). Other brain regions, such as the anterior cingulate cortex and dorsolateral prefrontal cortex, are crucial in monitoring conflicting information and expectation discrepancies, ultimately influencing future decisions (Li and Tracer, 2017). Fair behavior can be satisfying, activating our brain’s reward networks. Studies have found that neural activity in the ventromedial prefrontal cortex and ventral striatum increases when a participant donates money to their preferred charity. Additionally, some researchers argue that the Theory of Mind – the ability to maintain a mental model of others’ thoughts – also plays a role in fair behavior (Takagishi et al., 2010). To guide decision-making, the medial prefrontal cortex is proposed to integrate emotional, deliberative, and social information, especially when social interests conflict with self-interest. Fairness may thus be a strategic choice influenced by understanding others’ reactions to our behaviors.

In a series of studies conducted by Crockett and colleagues, the role of serotonin in shaping our reactions to fairness was investigated (Crockett et al., 2008, 2010a,b, 2013). The researchers found that participants with depleted serotonin levels were more likely to reject a greater proportion of unfair offers but not fair offers without showing changes in mood, judgment, basic reward processing, or response inhibition. On the other hand, enhancing serotonin levels made participants more likely to judge harmful actions as forbidden, but only in cases where the harms were emotionally salient. Furthermore, increasing serotonin levels in participants resulted in a decreased likelihood of rejecting unfair offers (i.e., the decision to decline a resource allocation they find inequitable, even if it comes at a personal loss; this action serves as a social deterrent against the proposer’s unfair behavior in future interactions). This implies that serotonin plays a crucial role in modulating specific retaliation rather than general norm enforcement. Participants with depleted serotonin were more inclined to punish unfair behavior directed toward themselves but not unfair behavior directed toward others. These findings highlight the significant influence of serotonin on our behavioral reactions to fairness and retaliation, shedding light on the complex neurobiological mechanisms that underlie our sense of justice and moral decision-making. This is especially relevant in the context of a growing mental health crisis, where disorders like depression, often linked to serotonergic dysfunction due to chronic stress, are increasingly prevalent. The implication is that individuals suffering from such disorders may be more sensitive to perceived injustices directed toward them but less responsive to unfairness affecting others.

At UCSF, a team of researchers led by Virginia Sturm has been investigating the impact of behavioral variant frontotemporal dementia (bvFTD) on prosocial choices and fairness behavior. BvFTD is a neurodegenerative disease characterized by atrophy in the frontal and temporal lobes, leading to significant changes in social and emotional functioning. In a study by Sturm et al. (2017), the researchers examined the relationship between prosocial deficits in bvFTD patients and the degree of atrophy in brain regions associated with reward processing. Utilizing a task that required participants to allocate money between themselves and others, the authors observed that bvFTD patients exhibited reduced prosocial behavior compared to healthy controls. Furthermore, this reduction in prosocial behavior was associated with atrophy in the ventromedial prefrontal cortex and the ventral striatum, both of which are key components of the brain’s reward network.

In a subsequent study, Sturm et al. (2018) explored the link between resting parasympathetic dysfunction and prosocial helping deficits in bvFTD patients. They found that bvFTD patients with decreased resting parasympathetic activity, as indicated by respiratory sinus arrhythmia, showed reduced prosocial behavior. This suggests impaired parasympathetic regulation might be linked to diminished prosocial tendencies in bvFTD patients. Moreover, these results align with research on heart rate variability (HRV) as a measure of parasympathetic activity. Notably, studies have shown a correlation between HRV and altruistic, prosocial behavior in the general population (Fooken, 2017). The findings suggest that HRV could serve as a modifiable biomarker for prosocial tendencies. Intriguingly, HRV can be improved through regular exercise and physical training, opening the door for potential interventions to enhance prosocial behavior. The implications of these findings could be far-reaching, warranting further research to explore HRV as a modifiable biomarker for prosocial behavior.

These studies provide insights into the neurological basis of “clinical unfairness” and highlight the need to understand bvFTD’s impact on brain areas related to social cognition and decision-making. This knowledge could guide the development of targeted interventions to address prosocial behavior deficits in such populations.

### Fairness and equity in brain health

Equity and fairness in healthcare are interconnected concepts that significantly contribute to advancing social justice and improving health outcomes for all, not just for specific subsets of individuals. This perspective is underscored by the research conducted by Dr. Ivan Arroyave, a notable contributor in the field and professor at the University of Antioquia, Colombia. In his research, Dr. Arroyave, who lectured in our salon, scrutinized the disparities in premature adult mortality in Colombia from 1998 to 2007, focusing on the correlation with educational attainment (Arroyave et al., 2014). The results indicated that individuals with only primary education were at a higher risk of premature death than those with post-secondary education. This study underscored the necessity for multi-sectoral policies to address these issues, especially among less educated populations, to enhance health equity.

Salon participants and hosts further reflected on the concept of brain health equity. The consensus was that fairness, when applied as a societal construct, could facilitate access to resources and opportunities that foster optimal brain health. The insights of Dr. Kai Kennedy, an Atlantic Fellow for Health Equity, Associate Professor, and Vice Chair of Equity at UCSF, further corroborated this viewpoint.

In a Q&A session during our salon, Dr. Kennedy highlighted strategies to address health disparities by promoting fairness and brain health equity. When asked about actions to prioritize in communities to address health inequity in relation to brain and mental health, Prof. Kennedy emphasized the importance of soliciting community perspectives, especially when forming partnerships with authoritative or external entities. She also stressed the need to understand local values, knowledge, and practices related to health and healthcare. Furthermore, she advocated for upholding sustainable collective self-determination as a goal, where community members can determine their ideal outcomes and play a significant role in defining interventions and assessing progress. When asked about a superpower that could address brain health inequity, Dr. Kennedy expressed a desire to see the invisible, illuminate issues in communities, and identify potential problem-solving resources and strategies. She noted that this superpower should not be exclusive; many people could have it and contribute unique perspectives to inform a collective consciousness and move toward community-identified solutions to relevant issues.

Equity and fairness in healthcare are interconnected concepts that significantly contribute to advancing social justice and improving health outcomes for all. While Dr. Kennedy’s insights provide a valuable framework for understanding the role of fairness in brain health equity, it is crucial to delve deeper into how fairness, as a societal construct, can translate into practical measures for equitable access to resources and opportunities for optimal brain health. Policy-making can be one of the most direct ways fairness impacts brain health equity, such as policies that ensure equitable access to healthcare resources like neuroimaging technologies or specialized neurological care. Fairness also extends to addressing the social determinants of health, like education, housing, and employment opportunities, which have a cascading effect on brain health. Community-based interventions, such as localized mental health programs, can be instrumental in promoting brain health equity, especially when they are culturally sensitive and tailored to meet the specific needs of a community. The advent of telemedicine and mobile health apps offers a unique opportunity to promote brain health equity by making healthcare more accessible, but it is crucial to ensure these technologies are equally accessible to all. Ethical considerations, particularly in the context of medical research, also play a role in ensuring that clinical trials for neurological conditions are inclusive and representative. By adopting a multifaceted approach that incorporates policy changes, addresses social determinants, and leverages community and technological resources, we can translate the societal construct of fairness into practical measures that promote brain health equity.

### Cultural perspectives on fairness and brain health equity

Our salon was conducted at UCSF, primarily involving a United States-based, highly educated, and possibly privileged demographic. However, it is crucial to note that the salon moderators and authors of this perspective come from diverse backgrounds, including Africa and Latin America. Moreover, Atlantic Fellows for Equity in Brain Health, most of whom hail from developing countries, participated in our activities, enriching the discussions with their personal and professional experiences.

One of the authors, an African-born, British-trained scientist, presented a compelling case study during the salon. She highlighted the stark disparities in brain health equity in Sub-Saharan Africa, a region often overlooked in global discussions. The case study touched upon the “Haves and Have Nots” in low- and middle-income countries, emphasizing the socioeconomic disparities within such nations. It also discussed the AFRICA-FINGERS project, aimed at addressing brain health inequity in low resource settings such as in Africa through a precision prevention framework (Kivipelto et al., 2020). The presentation described the African concept of Ubuntu, which emphasizes the interconnectedness of humanity, suggesting that fairness and equity are not just individual but collective responsibilities.

By incorporating these diverse viewpoints, we can better understand how fairness as a societal construct can translate into practical measures that promote equitable access to resources and opportunities for improving brain health globally. This inclusion of cultural perspectives also advances our understanding of fairness and its implications for brain health equity, thereby strengthening the overall impact of our work.

### The future of fairness

In today’s rapidly evolving and polarizing social landscape, concerns have arisen over the potential decline in fairness and prosocial behaviors. Prosocial tendencies, which involve acting in the best interest of others, are generally stronger with increased social closeness and tend to diminish as social distance grows. The rise of isolated social bubbles and echo chambers in contemporary society sparks concern regarding the implications for fairness and social cooperation. Social bubbles refer to the phenomenon in which individuals primarily associate with others who share similar values, beliefs, and opinions (Arguedas et al., 2022). This can result in a lack of exposure to diverse perspectives and reinforcement of existing biases. Additionally, echo chambers are environments where people are repeatedly exposed to the same viewpoints, causing a confirmation bias and an amplification of those opinions (Brugnoli et al., 2019). Both social bubbles and echo chambers can be perpetuated by social media algorithms that prioritize content that aligns with users’ preferences, further exacerbating the issue.

The social consequences of social bubbles and echo chambers can be detrimental to fairness and cooperation. As individuals become more entrenched within these environments, they may increasingly perceive those outside their social circles as strangers or even as adversaries. Consequently, empathy, understanding, and concern for the well-being of others may decline, resulting in decreased motivation to treat others fairly. In a more divided society, this trend can contribute to increased polarization, reduced tolerance, and heightened intergroup conflicts.

The possibility of declining fairness in society raises essential questions about whether we can reverse this trend. As discussed above, if heart rate variability (HRV) is a biomarker for prosocial behavior, interventions aimed at improving it could have broader societal implications. For instance, regular exercise has been shown to improve HRV, suggesting a hypothetical strategy for fostering prosocial behavior (De Couck et al., 2019). This aligns with the holistic notion of “healthy body, healthy mind, healthy societies.” While this is admittedly a long shot and requires further empirical validation, the idea presents an intriguing avenue for future research and potential community-based interventions.

Another approach might be trying to effectively enhance empathy, a critical factor in promoting fairness. Empathy is a complex construct, encompassing affective, cognitive, and intentional components (Decety and Yoder, 2016). While modifying affective empathy may be challenging, research suggests that cognitive empathy and empathic concern could be more promising targets for intervention. A study by Decety and Yoder (2016) examined the relationship between different aspects of empathy and sensitivity to justice for others, as well as the endorsement of moral rules. They found that cognitive empathy and empathic concern, rather than emotional empathy, predicted participants’ sensitivity to justice and moral rule endorsement. Cognitive empathy involves the ability to understand another person’s perspective and feelings, while empathic concern is the capacity to feel concern and compassion for others. On the other hand, emotional empathy refers to sharing another’s emotional experience.

These findings, coupled with the overall contributions from the GBHI Fairness Salon 2023, suggest that to foster fairness in society that could have a direct impact and/or lead to equitable brain health outcomes, efforts should focus on promoting perspective-taking, reasoning, and empathic concern, rather than solely emphasizing emotional sharing with those experiencing misfortune. In other words, to enhance fairness, we must actively reflect on the concept and intentionally cultivate it as a core value.

## Data availability statement

The original contributions presented in the study are included in the article/Supplementary material, further inquiries can be directed to the corresponding author.

## Author contributions

All authors listed have made a substantial, direct, and intellectual contribution to the work and approved it for publication.
