# Supplementary material for: Fairness: from the guts to the brain – a critical examination by Atlantic fellows of the Global Brain Health Institute
Source: Front Psychol. 2023 Oct 19;14:1241125. doi: 10.3389/fpsyg.2023.1241125 (PMC10620796; doi:10.3389/fpsyg.2023.1241125)
Supplement: Supplementary file 1 [file Data_Sheet_1.docx]

**Supplementary Appendix A: Visual documentation of the social experiment game interface.**

**
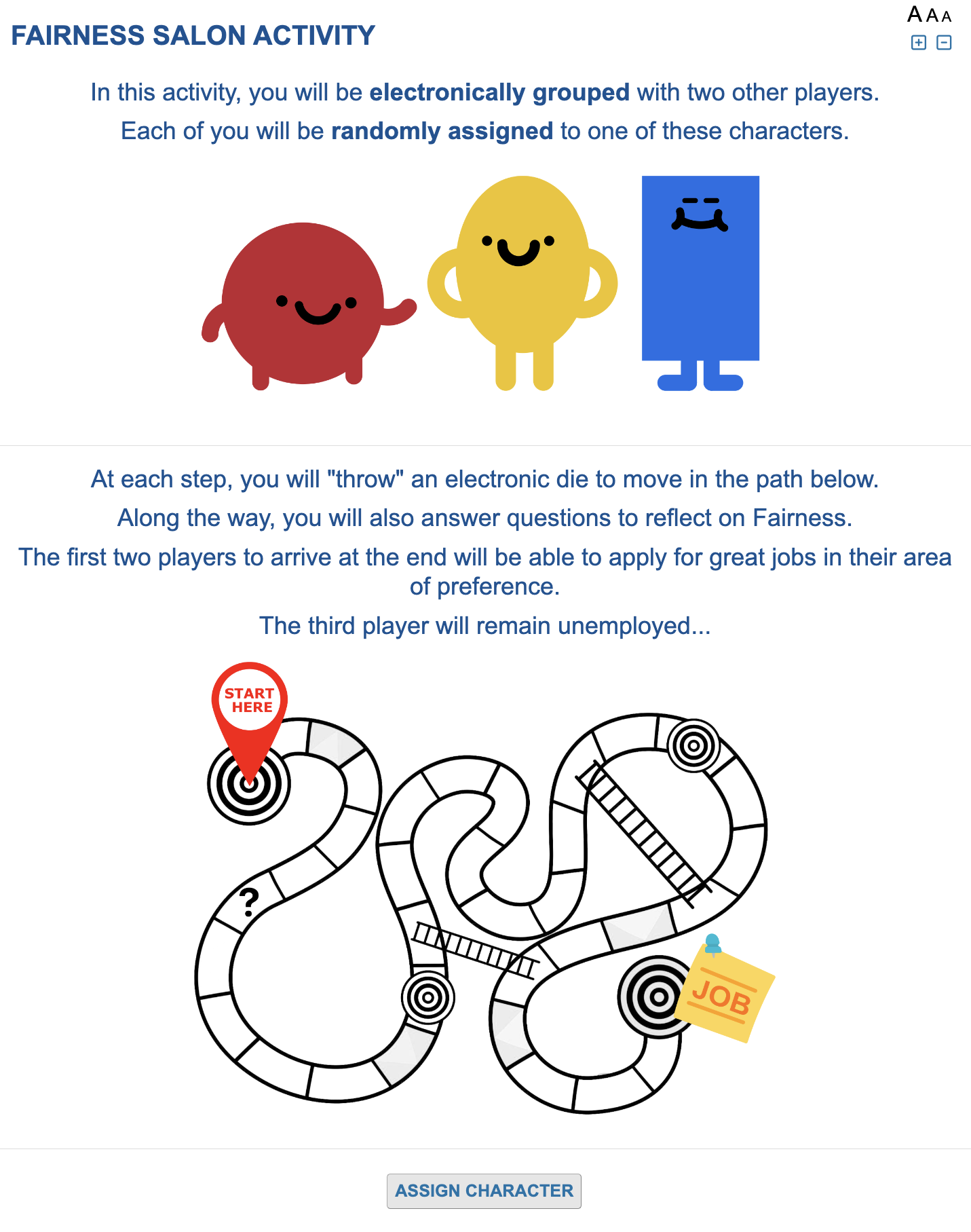
**

**Step 1: Introduction**

This screen introduced the activity and its objectives. Participants were informed that they would be grouped with two other players and engage in a game related to fairness. While they were prompted to answer questions to progress, these responses did not influence the game's outcome. Instead, they were collected to enrich subsequent discussions in the online salon.

**
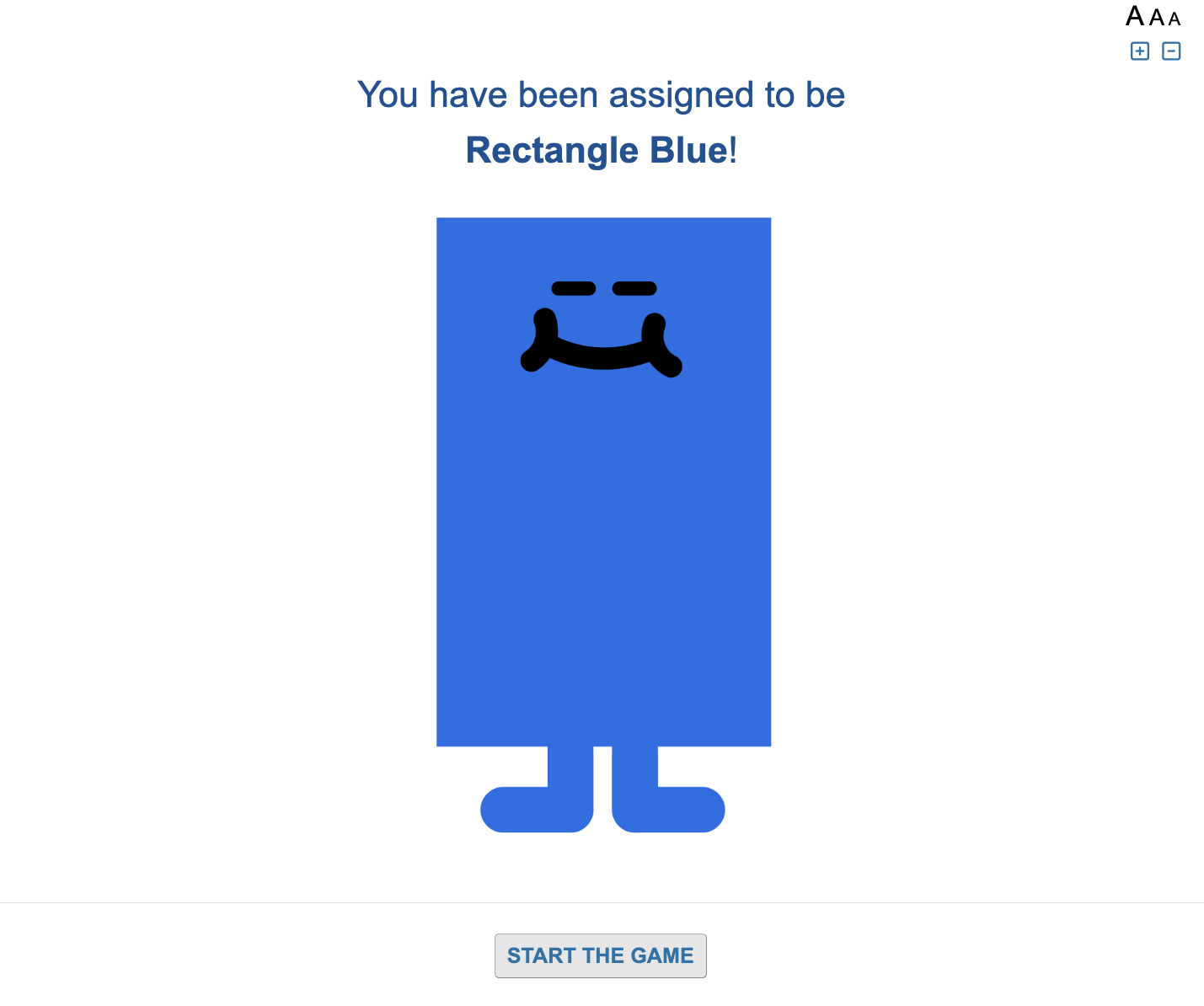
**

**Step 2: Character Assignment**

Participants were assigned a character for the game. Without their knowledge, all participants were assigned to the same character (i.e., the yellow and red characters were computer non-player characters).

**
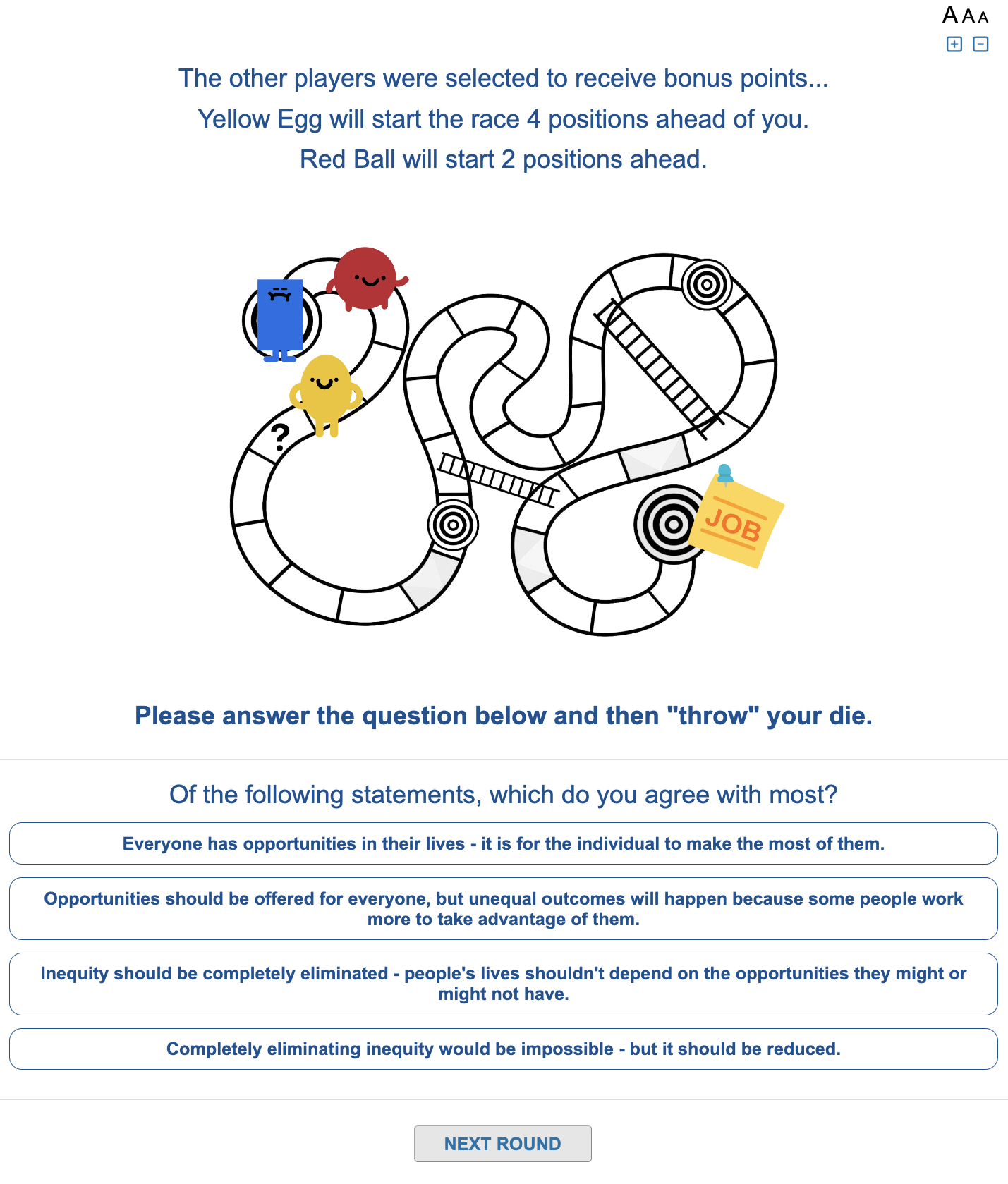
**

**Step 3: Game Start and Initial Conditions**

This screen set the initial conditions for the game, including any advantages or disadvantages for the players. It also presented the first question related to fairness for participants to answer before proceeding by "throwing" their electronic die.

**
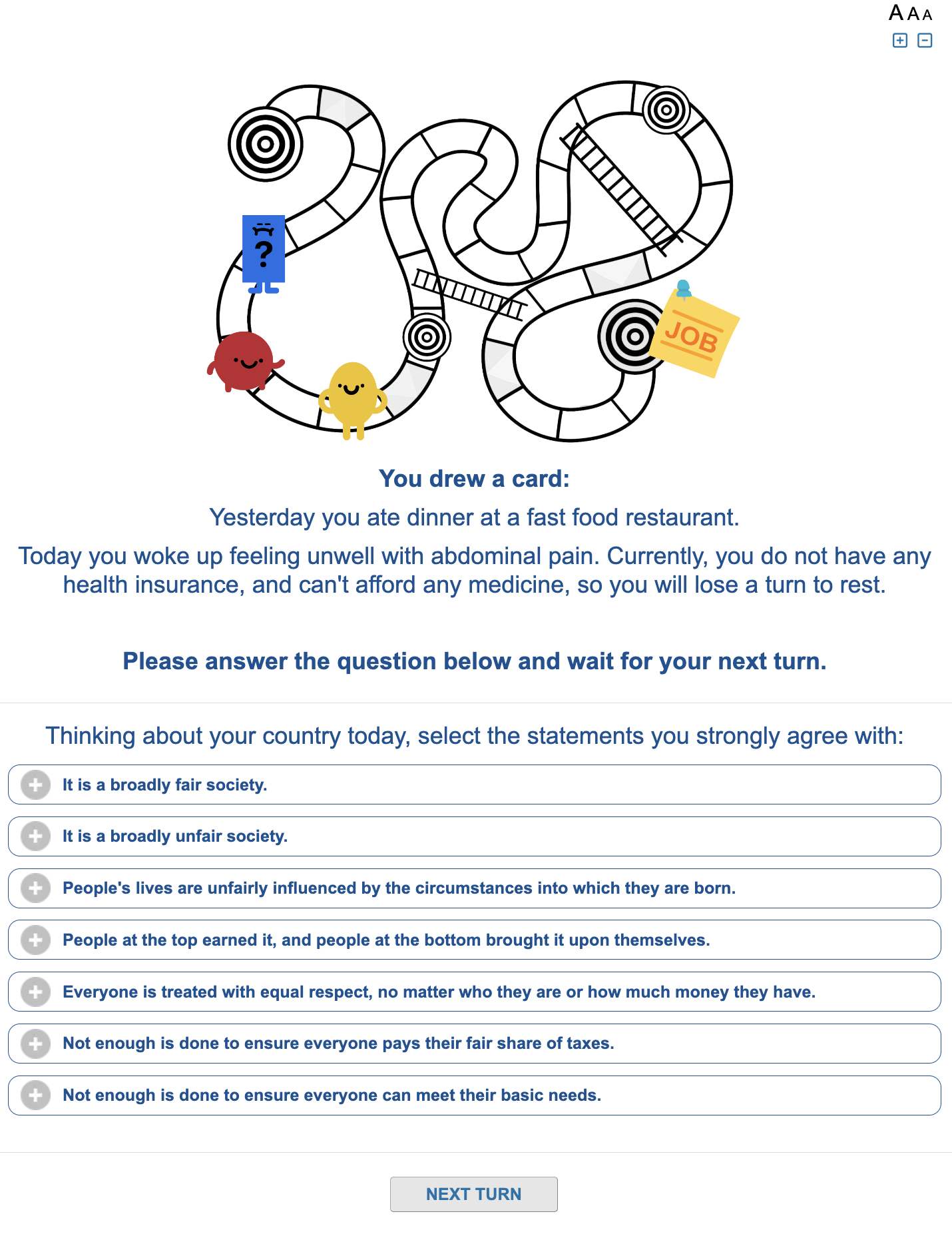
**

**Step 4: Event Card - Health Issue**

This screen presented a scenario where the participants faced a health issue, affecting their progress in the game. Another question about societal fairness was posed.

**
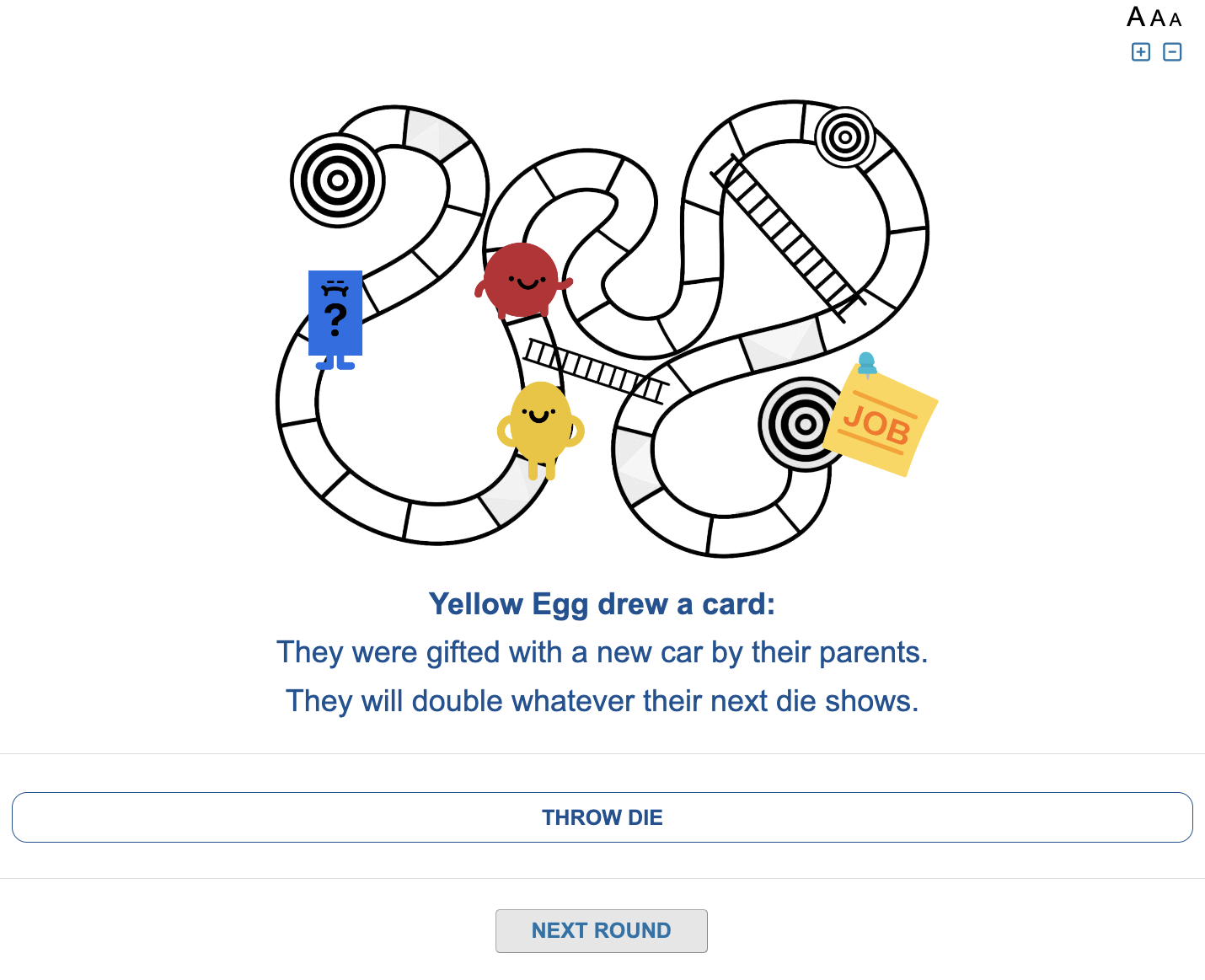
**

**Step 5: Event Card - Yellow Egg's Advantage**

This screen showed an event card that benefitted the Yellow Egg character, further altering the game dynamics.

**
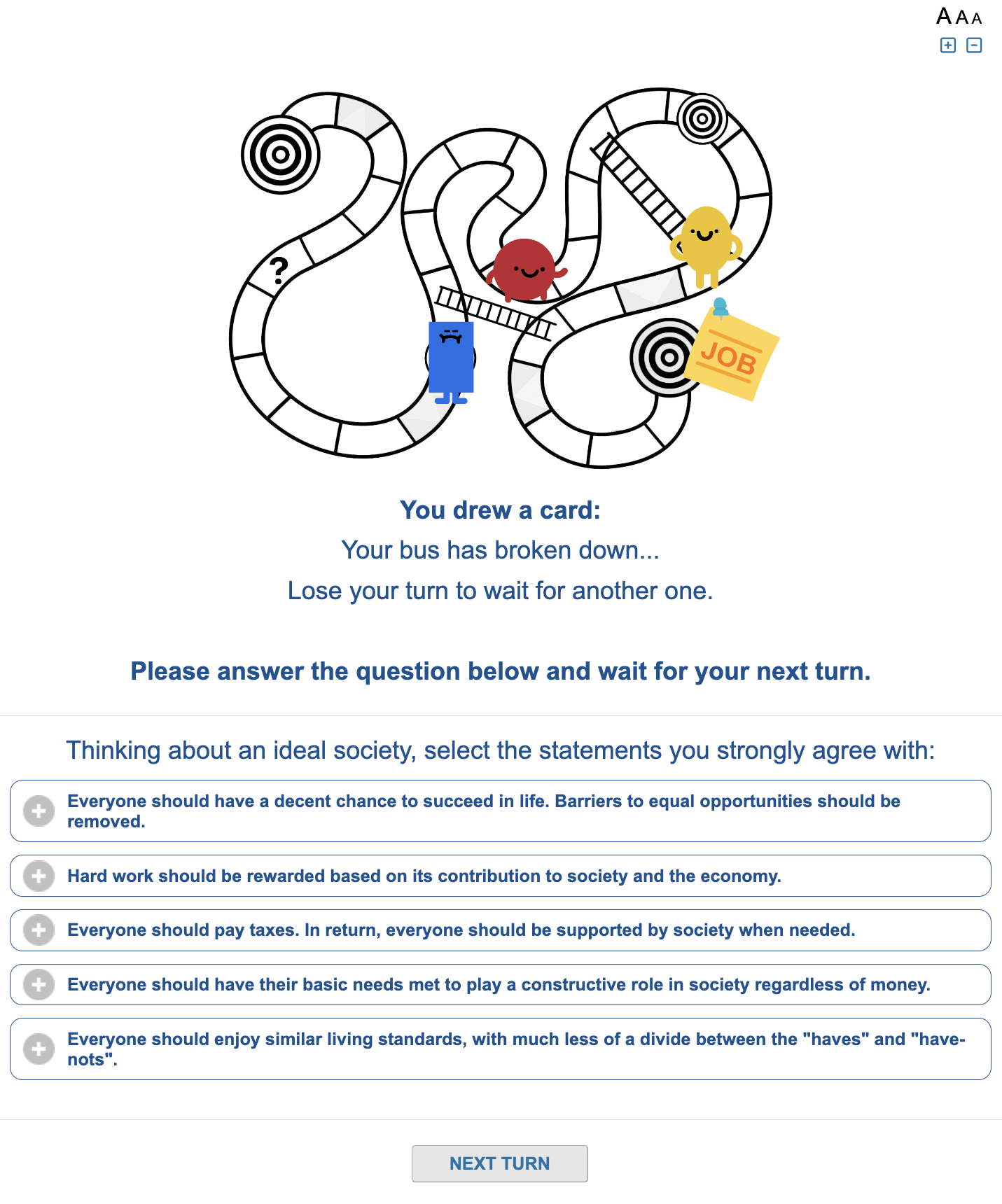
**

**Step 6: Event Card - Transportation Issue**

The participants encountered a transportation issue, causing a delay in their game progress. Another question about an ideal society was presented.

**
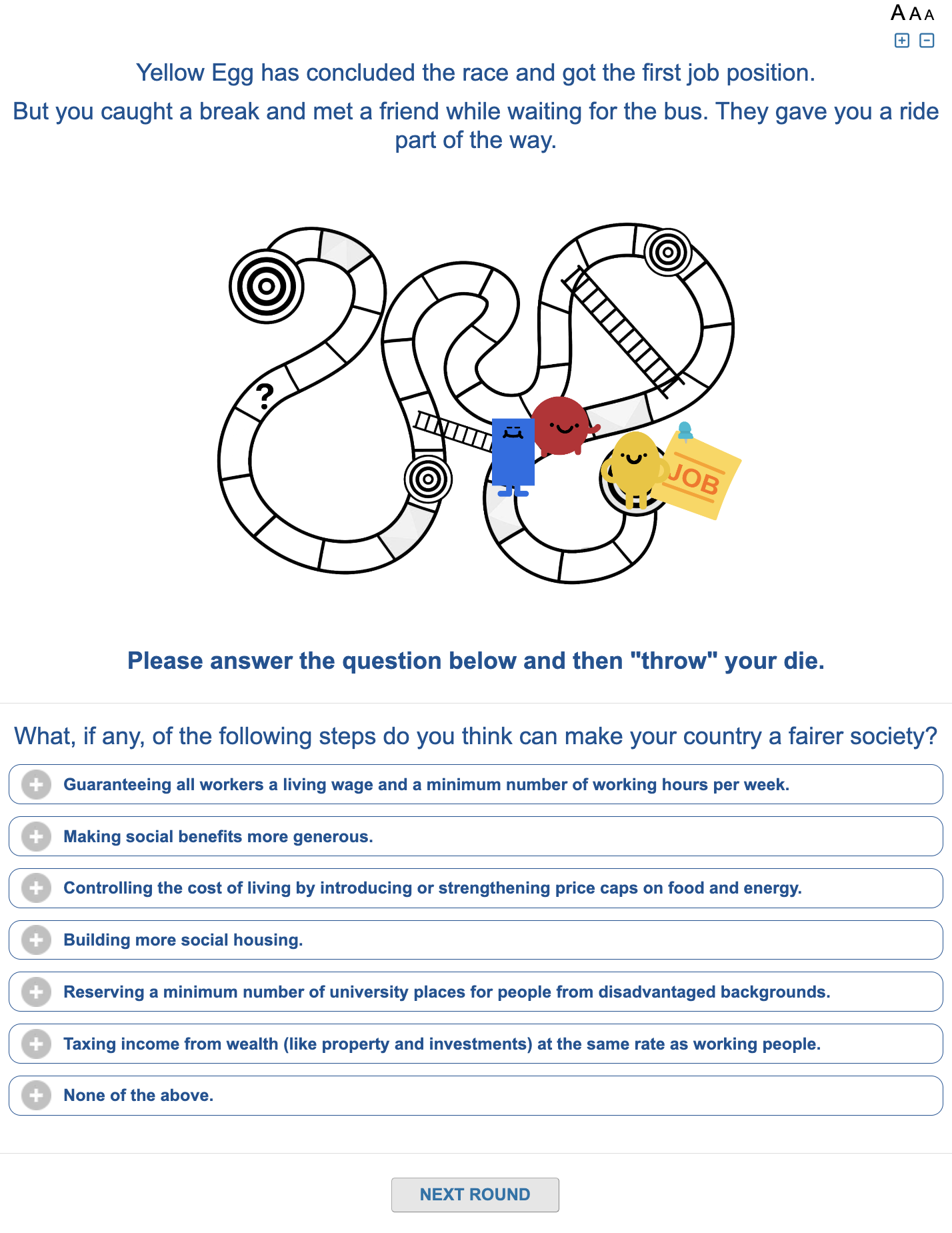
**

**Step 7: Yellow Egg's Success and Participant's Progress**

This screen showed that Yellow Egg secured the first job position. The participants also received help from a friend, affecting their game progress. Another question about societal fairness was posed.

**
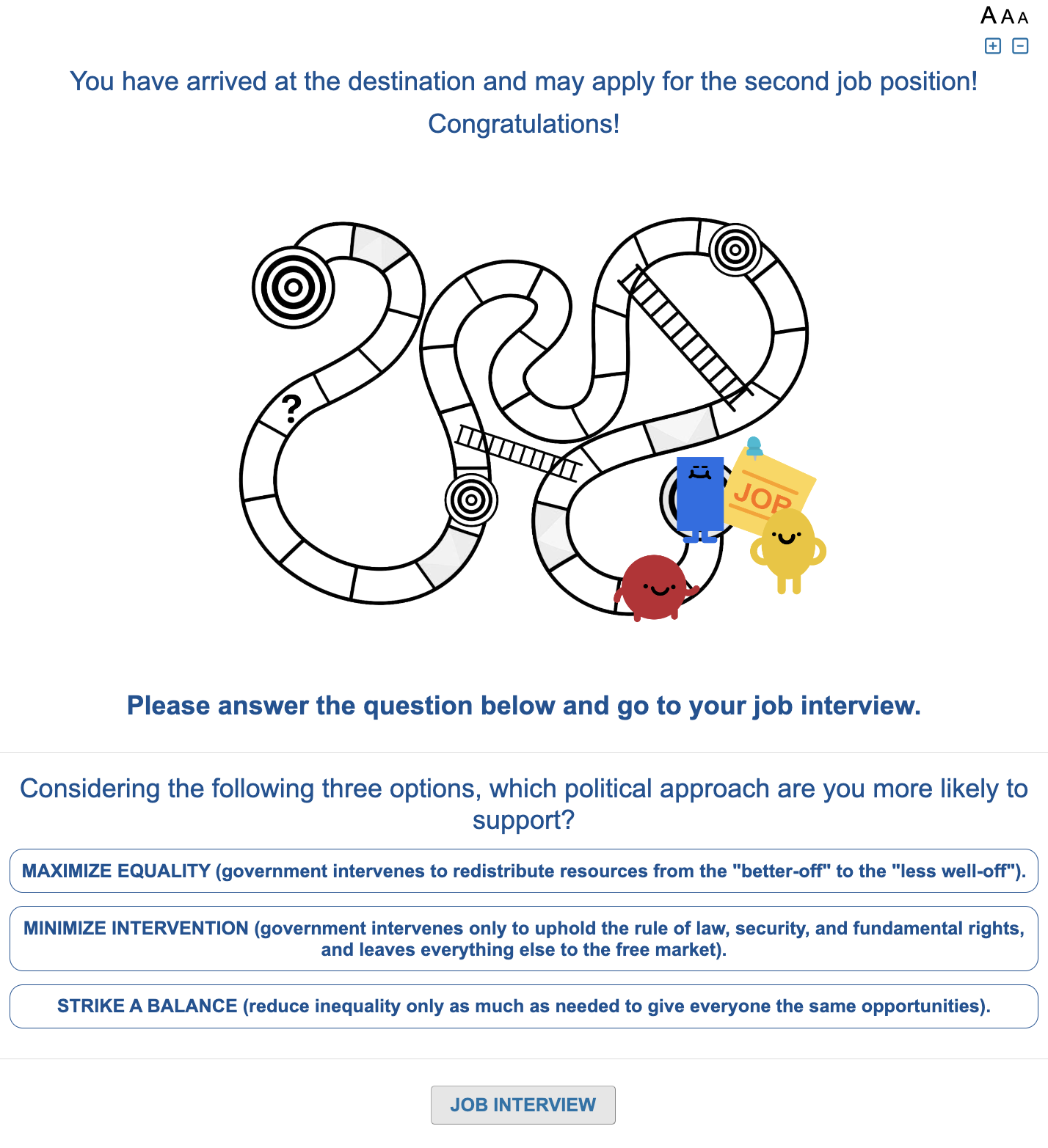
**

**SCREEN 8: Reaching the Destination**

The participants reached the end of the game and were given the opportunity to apply for the second job position. A final question about political approaches to fairness was presented.

**
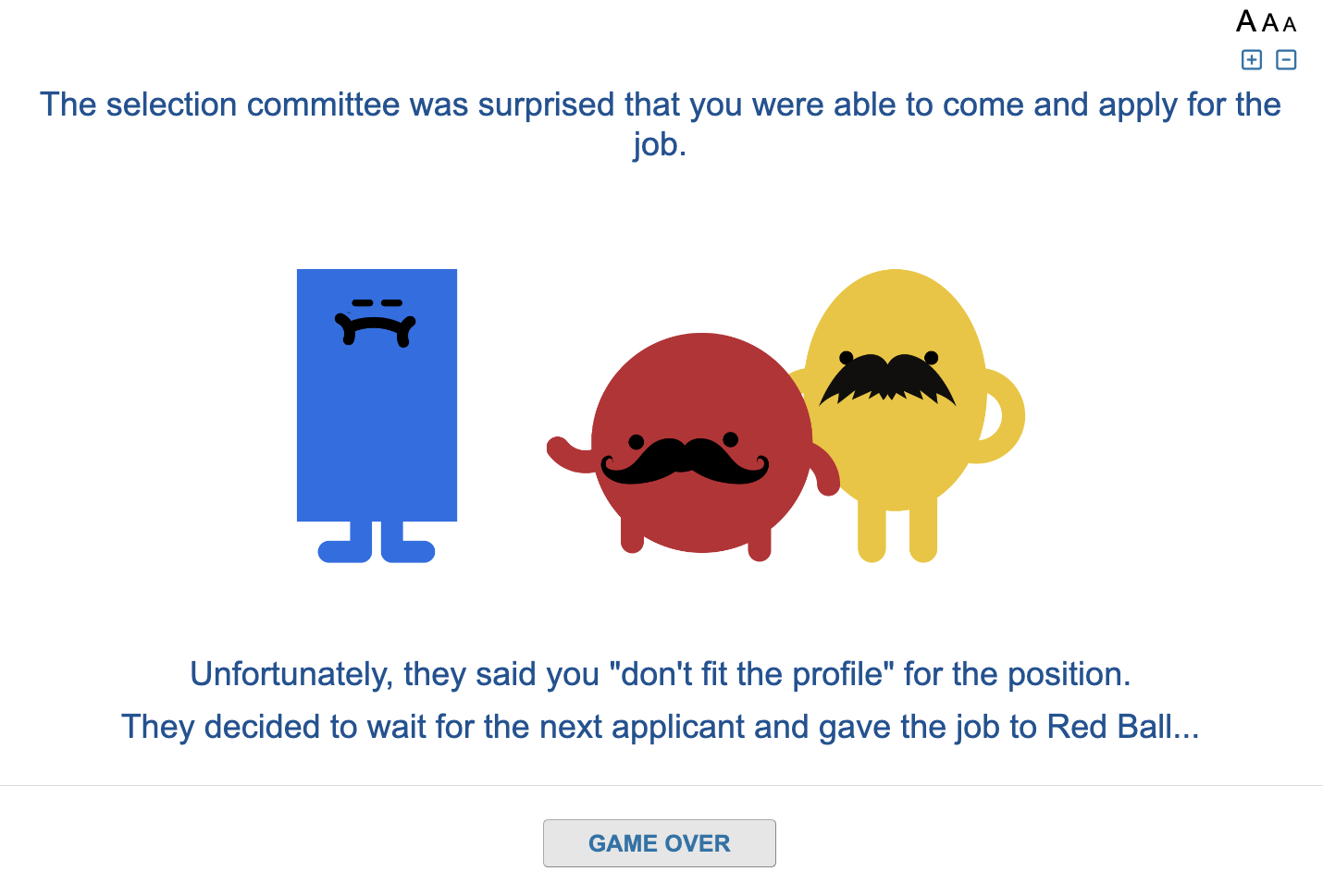
**

**Step 9: Job Interview Outcome**

This screen revealed the outcome of the job interview, indicating whether the participant succeeded in securing the job.

**
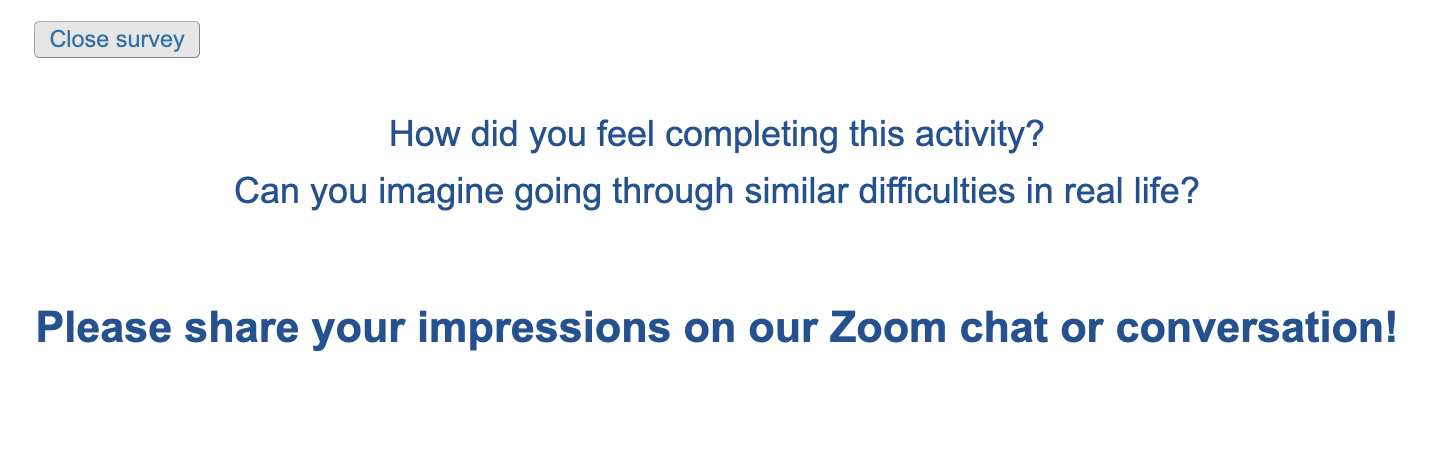
**

**Step 12: GAME OVER**

The activity concluded by asking participants to explore their feelings and impressions of the game.
